# A Comparative Analysis of the Antioxidant Profiles Generated by the RoXsta^TM^ System for Diverse Biological Fluids Highlights the Powerful Protective Role of Human Seminal Plasma

**DOI:** 10.3390/antiox14010090

**Published:** 2025-01-14

**Authors:** Robert J. Aitken, Alexandra Wilkins, Natasha Harrison, Mohammad Bahrami, Zamira Gibb, Kaitlin McIntosh, Quan Vuong, Sarah Lambourne

**Affiliations:** 1 Centre for Reproductive Science, University of Newcastle, Newcastle, NSW 2308, Australia; alex.wilkins@newcastle.edu.au (A.W.); natasha.harrison@newcastle.edu.au (N.H.); mo.bahrami@newcastle.edu.au (M.B.); zamira.gibb@newcstle.edu.au (Z.G.); kaitlin.mcintosh@uon.edu.au (K.M.); sarah.lambourne@newcastle.edu.au (S.L.); 2 Hunter Medical Research Institute, Newcastle, NSW 2305, Australia; 3 School of Environmental and Life Sciences, University of Newcastle, Brush Rd., Ourimbah, NSW 2258, Australia

**Keywords:** antioxidant, RoXsta^TM^ system, semen, sperm motility, blood, urine, saliva, follicular fluid, fruit juices

## Abstract

(1) Background: The RoXsta^TM^ system has been developed as a rapid, effective means of profiling different types of antioxidant activity. The purpose of this study was to examine its performance utilizing a diverse array of biological fluids including semen, blood plasma, serum, urine, saliva, follicular fluid and plant extracts. (2) Methods: The RoXsta^TM^ system was used to assess the ability of different fluids to suppress free radical formation as well as scavenge a variety of toxic oxygen metabolites including free radicals and both hydrogen and organic peroxides. (3) Results: Human semen was shown to have significantly (*p* < 0.001) more peroxide scavenging power than any other fluid tested (10–14 mM vitamin C equivalent compared with 1–2 mM for blood serum or plasma), while urine was particularly effective in scavenging free radicals and preventing free radical formation (*p* < 0.001). The powerful antioxidant properties of human semen were shown to reside within the seminal plasma (SP) fraction, rather than the spermatozoa, and to be resistant to snap freezing in liquid nitrogen. Moreover, comparative studies demonstrated that human SP exhibited significantly (*p* < 0.001) higher levels of antioxidant potential than any other species examined (stallion, bull, dog) and that this intense activity reflected the relative vulnerability of human spermatozoa to peroxide attack. (4) Conclusions: The RoXsta^TM^ system provides valuable information on the antioxidant profile of complex biological fluids, supporting its diagnostic role in conditions associated with oxidative stress. Based on the results secured in this study, human semen is identified as a particularly rich source of antioxidants capable of scavenging both hydrogen and organic peroxides, in keeping with the high susceptibility of human spermatozoa to peroxide-mediated damage.

## 1. Introduction

A wide range of human and animal pathologies are thought to be related to the development of oxidative stress including cancer, neurodegenerative conditions, depression, multiple sclerosis, amyotrophic lateral sclerosis, cataracts, diabetes, cardiovascular disease and infertility [1,2]. Such associations have led to intense interest in the use of antioxidant supplements to restore the balance between reactive oxygen species (ROS) production and antioxidant protection. Unfortunately, the results of clinical trials addressing this topic have been variable and, ultimately, disappointing [3,4]. Apart from the pharmacological properties of the antioxidant agents themselves, one of the major issues with such evaluations relates to the selection of appropriate patients for treatment. Many clinical trials involving antioxidants have failed to select patients that are actually suffering from detectable oxidative stress and might reasonably be expected to benefit from antioxidant supplementation. In the field of infertility research, for example, males have been selected for such treatment because they exhibited oligozoospermia (low sperm numbers), asthenozoospermia (poor sperm motility), teratozoospermia (poor sperm morphology), high levels of DNA fragmentation, varicocele, prolonged idiopathic subfertility or recurrent pregnancy loss [4,5,6,7,8,9,10,11,12], rather than any direct evidence of heightened oxidative stress. Under these circumstances, the results, not surprisingly, have been inconclusive because the aetiologies underpinning these reproductive conditions are complex and, in some cases, may not involve any element of redox imbalance [13]. Furthermore, administering powerful antioxidants to patients who are not antioxidant deficient brings with it a risk of creating reductive stress, which can be just as harmful as its oxidative counterpart [14].

With such poorly selected patient populations, it is entirely predictable that clinical trials addressing the value of antioxidants in the treatment of conditions such as male infertility will generate variable results; some patients will get better, some will get worse and, overall, any therapeutic benefit will just become lost in the statistical noise. On the rare occasion that such patients have actually been selected on the basis of verifiable oxidative stress, then the application of antioxidants has been found to generate positive clinical outcomes [15]. Thus, before we spend any more time and resources on clinical trials investigating the benefits of antioxidant therapy for infertility, or any other kind of pathology, we need to develop simple robust methodologies to test for the oxidative stress we are trying to counter [16].

To address this need, we have recently developed a novel suite of assays (the RoXsta^TM^ system) to measure different aspects of antioxidant activity using ABTS [2,2′-azino-bis (3-ethylbenzothiazoline-6-sulfonic acid)] as a redox sensor [17]. These assays were developed to measure the ability of biological fluids to suppress free radical formation as well as engage in the scavenging of free radicals, hydrogen peroxide and organic peroxides. Using human seminal fluid to evaluate the biological significance of these assays, we found that their combined output correlated well with such physiological parameters as sperm count, motility, the generation of ROS by the sperm mitochondria, sperm DNA damage and the presence of cytotoxic lipid aldehydes (specifically malondialdehyde) in semen [17]. The purpose of the present study was to examine the antioxidant profiles generated by these assays in a range of biological materials including blood plasma, serum, urine, saliva, follicular fluid and semen. The results confirm the capacity of these assays to provide rapid, effective assessments of antioxidant potential in a wide variety of contexts and highlight the particularly powerful protective properties of human seminal plasma.

## 2. Materials and Methods

### 2.1. Reagents and Suppliers

Unless otherwise stated, all reagents were purchased from Sigma Aldrich (St. Louis, MO, USA).

### 2.2. Human Sample Preparation

All human experiments were conducted following the protocols approved by the University of Newcastle Human Research and Ethics Committee and the State Government (H-2013-0319, H-2023-0385 and 200621). Semen samples were obtained from healthy unselected male donors following 2–3 days of sexual abstinence and delivered to the laboratory within 1 h of ejaculation. Whenever seminal plasma (SP) was required, the semen was centrifuged for 5 min at 500× *g* and the plasma removed, either to be analysed fresh or after freezing in liquid nitrogen and storage at −80 °C. Whenever spermatozoa were required, the semen was processed through Percoll gradients as previously described [18] and the high-density fraction resuspended in HEPES-buffered Biggers–Whitten–Whittingham medium (BWW) supplemented with 1 mg/mL polyvinyl alcohol at a concentration of 10 × 10^6^/mL. In addition to spermatozoa, saliva samples were collected from randomly selected individuals and analysed fresh without freezing.

### 2.3. Equine Sample Preparation

Institutional and New South Wales State Government ethical approval was secured for the use of equine material in this study (ACEC number A-2011-122). Equine semen was collected from normozoospermic Shetland and miniature crossbred pony stallions of proven fertility using a Missouri artificial vagina (AV; Minitube, Ballarat, VIC, Australia). Semen was centrifuged (5 min at 500× *g*), and SP was collected. Blood was obtained with vacutainers using either a clotting tube for serum formation or a green-topped heparin tube for blood plasma. Tubes were centrifuged (10 min at 1000× *g*) either immediately or 10–15 min after collection in the case of serum, and the resulting supernatants frozen and stored at −80 °C.

### 2.4. Bovine Sample Preparation

Institutional and New South Wales State Government ethical approval was secured for the use of bovine material in this study (ACEC number A-2022-223). The bulls utilized in this study were confined to a crush for semen collection. After a rectal examination to clear the anus of any excess faecal matter, a water-based, non-spermicidal lubricant was applied to a rectal probe (75 mm to 90 mm) and inserted into the anal cavity. Pulses from an electro-ejaculation unit were rhythmically applied (every 2–3 s, with 1 s interval breaks) until ejaculation occurred. A silicon funnel with a 15 mL Falcon tube attached was used to collect the ejaculate. The raw ejaculate was centrifuged (400× *g*) for 20 min, and the SP collected, snap frozen and stored at −80 °C. Cow ovaries were obtained from a local abattoir and transported back to the laboratory in prewarmed 0.9% saline solution (35–38 °C). Antral follicles were subsequently aspirated using an 18-gauge needle attached to a 10 mL syringe and the resulting follicular fluid snap frozen in liquid nitrogen and stored at −80 °C. In similar fashion, an 18-gauge needle was inserted into the bladder to allow the collection of post-mortem urine specimens, which were subsequently snap frozen and stored at −80 °C.

### 2.5. Canine Sample Preparation

Canine semen was kindly collected and donated by an accredited breeder registered with Dogs NSW (Kerensa Kennels, Dogs NSW registration number 2100076617). Three healthy male dogs, 4–9 years old, were used for the semen collections. The breeds were an Australian Kelpie and two Border Collie dogs. The included ejaculates had >80% morphologically normal spermatozoa and a motility of >80%. From each dog, the sperm-rich fraction of the ejaculate was collected in a calibrated, plastic Falcon tube by digital manipulation [19]. A portion of each ejaculate was subsequently transferred to Eppendorf tubes and centrifuged at 500× *g* for 5 min. The SP was then carefully collected from above the sperm pellet and transferred into clean tubes in 500 µL aliquots before being frozen and stored at −80 °C.

### 2.6. Impact of Cumene Hydroperoxide on Sperm Motility

In order to determine whether the observed interspecies differences in levels of antioxidant protection provided by SP reflected the vulnerability of the spermatozoa to oxidative stress, time- and dose-dependent studies were conducted on the impact of cumene hydroperoxide on sperm motility. For this study spermatozoa from different species were prepared on discontinuous density gradients and the isolated spermatozoa suspended in a medium at a concentration of 10 × 10^6^/mL in BWW. Spermatozoa were then treated with various doses of cumene hydroperoxide (1, 0.5, 0.25, 0.125, 0.06 and 0 mM), and after two time points (15–30 min and 120–135 min), both total motility(average path velocity of >5 µm/s) and progressive motility (straightness of >80% and average path velocity of >25 µm/s) were assessed using a CASA (Computer-Aided Sperm Analysis) system (Hamilton Thorne, IVOS II, Beverly, MA, USA) and parameter settings that had been optimized for each species assessed.

In order to confirm the protective properties of human seminal plasma, Percoll-purified suspensions of human spermatozoa, at a concentration of 10 × 10^6^/mL, were exposed to a fixed dose of cumene hydroperoxide (0.25 mM) in the presence or absence of varying amounts of human seminal plasma (12.5%, 6.25%, 3.125% and 0%). The impact of this treatment on motility was then determined using the above CASA system after 15–30 min exposure.

### 2.7. The Antioxidant Assay System

The RoXsta^TM^ (Rapid oXidative stress assays) antioxidant system used in these studies has been previously described in detail [17]. In essence, this assay uses ABTS as a redox active probe and measures 4 different kinds of antioxidant activity. (1) Organic peroxide scavenging activity, assessed by determining the ability of a given fluid to suppress formation of the ABTS•^+^ radical using a reaction mixture containing 675 µL phosphate buffer, 25 µL ABTS (250 µM), 100 µL diluted sample and 100 µL hematin (0.05 mg/mL) at pH 6.5. The reaction was activated by the addition of 100 µL and 1 mM cumene hydroperoxide (100 µM final concentration) and incubated at room temperature for 20 min before the absorbance was determined at 734 nm in a spectrophotometer (SPECTROstar Nano, BMG Labtech, Ortenberg, Germany). (2) Hydrogen peroxide scavenging activity, assessed by determining the ability of a given fluid to suppress formation of the ABTS•^+^ radical in a reaction mixture comprising 735 µL buffer, 15 µL ABTS (150 µM), 100 µL diluted sample and 50 µL horse radish peroxidase (HRP; 0.05 mg/mL). In this case, the reaction was activated by the addition of 100 µL hydrogen peroxide (30 µM final concentration) and incubated at room temperature for 10 min to allow formation of the coloured ABTS•^+^ radical cation which was then read at 734 nm. (3) Free radical scavenging activity was assessed by determining the ability of a given fluid to scavenge the ABTS•^+^ cation radical prepared by the oxidation of ABTS (100 µM) in phosphate buffer (pH 4.8) within the anodic chamber of an electrochemical cell [17]. For this post-activation assay, 1 mL of activated ABTS•^+^ was removed to a 1.5 mL Eppendorf tube and the suppression of absorbance measured at 734 nm after 5 min after addition of the test sample in 15 µL. (4) Inhibition of free radical formation in a pre-activation assay in which 100 µL of the diluted sample was added to 5.4 mL 100 µM ABTS at pH 4.8. A 2 mL aliquot of this mixture was then added to the anodic chamber of the electrochemical cell. Current (20 sec at 45 V and 10 mA) was subsequently applied resulting in the appearance of ABTS•^+^ at the anode, the cathodic chamber serving as a passive control for sample turbidity. Following the passage of current, the anodic and cathodic chambers were agitated and then left for 5 min to allow the activation to proceed to completion and the suppression of ABTS•^+^ formation to be determined. For all of these antioxidant assays, vitamin C (ascorbic acid) was used as a positive control and the results are expressed as vitamin C equivalents. Three repeat analyses were conducted on at least 3 independent biological replicates for each of the materials assessed in this study.

For each assay, optimization studies were conducted to determine the sample dilution at which the various assessments of antioxidant activity should be conducted. The results of this analysis are presented in Supplementary Table S1.

### 2.8. Statistics

Statical analysis of the data was undertaken using the JMP Pro 17 statistical software package (SAS Institute, Cary, NC, USA). All data are presented as the mean ± the standard error (S.E.M.) of at least 3 independent replicates. The data were initially screened using the Shapiro–Wilk test to determine the normality of data distribution. Data that did not conform to a normal distribution were transformed using the Box Cox transformation with optimized lambda values calculated for each parameter. In the Figures, all transformed data are presented in their original form for easier interpretation. Differences between groups were determined by ANOVA with post hoc analysis utilizing Tukey’s honest significant difference test (HSD). Throughout, the minimum level of statistical significance was set at *p* < 0.05.

## 3. Results

### 3.1. Impact of Spermatozoa and Freezing on the Antioxidant Activity of Human Semen

**Figure 1:**
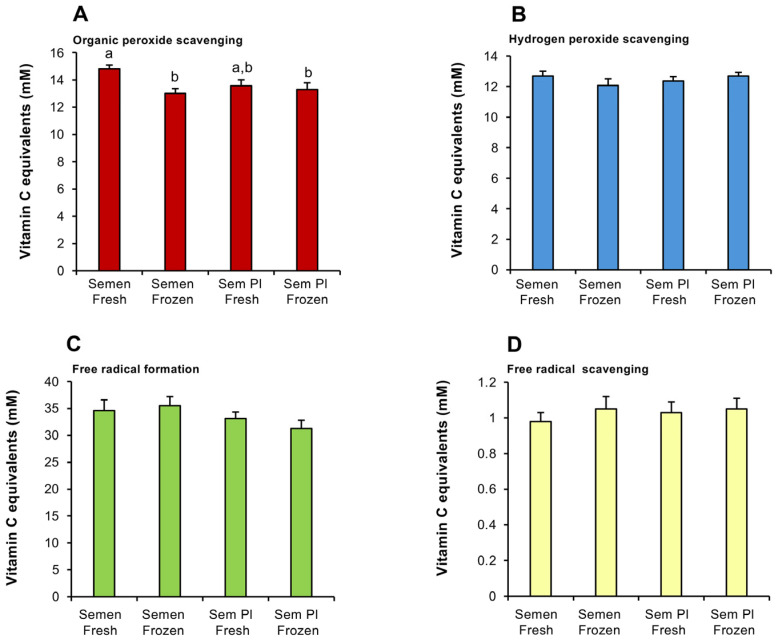
Impact of sperm presence and freezing on the antioxidant activity of human semen. (**A**) Organic peroxide scavenging activity. (**B**) Hydrogen peroxide scavenging activity. (**C**) Inhibition of ABTS•^+^ radical formation. (**D**) ABTS•^+^ radical scavenging activity. All results are expressed as vitamin C equivalents. All columns not connected by the same letter are significantly different. Data presented as means ± S.E.M.; (*n* = 9).

This analysis was initiated by an assessment of human semen samples to determine the levels of antioxidant activity expressed, and the extent to which they would be influenced by the presence or absence of spermatozoa and/or being frozen in liquid nitrogen (Figure 1A–D). The results indicated that for three of the antioxidant activities assessed (hydrogen peroxide scavenging, free radical scavenging and the suppression of free radical formation), the performance of human semen was neither impacted by the presence of spermatozoa nor being snap frozen in liquid nitrogen (Figure 1B–D). However, the organic peroxide scavenging assay revealed a modest decrease (*p* < 0.01) when semen was frozen and a slight reduction when spermatozoa were removed that bordered statistical significance (*p* = 0.055) (Figure 1A).

Together, these results suggested that live spermatozoa make a minor contribution to the antioxidant capacity of human semen; a vast majority of the antioxidant activity was contained within the SP fraction and did not change significantly following freezing (Figure 1A). The dominant form of antioxidant activity in semen involved the capacity of this fluid to suppress free radical formation (~30–35 mM vitamin C equivalents), followed by organic peroxide scavenging (~13–15 mM vitamin C equivalents), hydrogen peroxide scavenging (~12–14 mM vitamin C equivalents) and free radical scavenging (0.75–1.35 mM vitamin C equivalents) activities (Figure 1A–D).

### 3.2. Antioxidant Activity of Semen Relative to Other Biological Fluids

In order to determine whether seminal plasma has evolved to provide spermatozoa with particularly high levels of antioxidant protection in relation to other biological fluids, the RoXsta^TM^ system was used to undertake a comparative analysis of the antioxidant profiles expressed by blood, urine, saliva, ovarian follicular fluid and semen.

**Figure 2:**
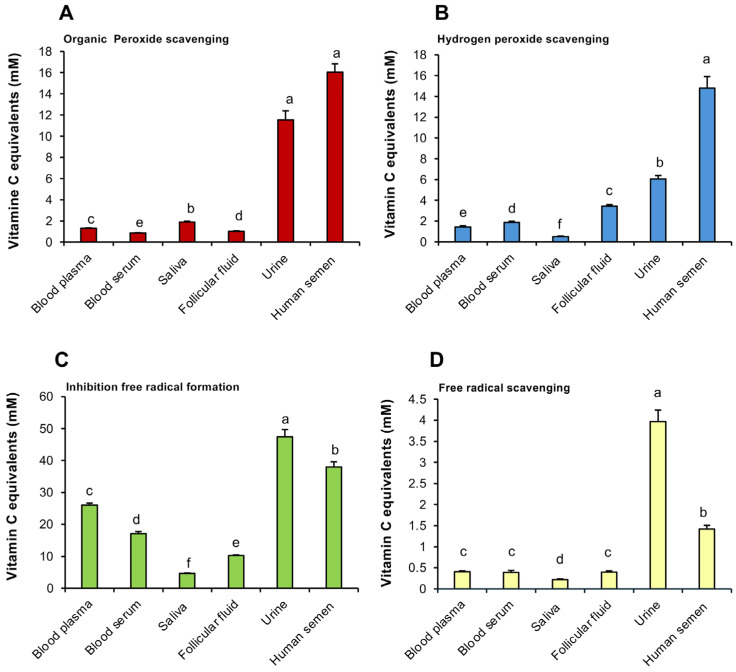
Analysis of antioxidant activity in a range of biofluids using the RoXsta^TM^ system. (**A**) Organic peroxide scavenging activity. (**B**) Hydrogen peroxide scavenging activity. (**C**) Inhibition of ABTS^•+^ radical formation. (**D**) ABTS^•+^ radical scavenging activity. All results are expressed as vitamin C equivalents. All columns not connected by the same letter are significantly different. Data presented as means ± S.E.M.; *n* = 9.

The results of this analysis revealed interesting differences between fluids in the levels of antioxidant protection afforded, as well as the types of antioxidant activity expressed (Figure 2A–D). With all four measures of antioxidant activity, human semen was significantly more active than any other fluid examined (*p* < 0.001) apart from urine, which was more active in scavenging free radicals and in suppressing the latter’s formation (Figure 2C,D). By contrast, follicular fluid possessed significantly lower levels of antioxidant activity than semen suggesting that the ovarian follicle does not confer the same protection to oocytes as seminal plasma affords to spermatozoa (Figure 2A–D). Both blood serum and plasma exhibited low levels of antioxidant protection relative to urine and semen in all assays (*p* < 0001). Importantly, however, some differences were observed between blood serum and plasma that might influence the way in which the RoXsta^TM^ system is applied in a clinical setting since the optimum sampling strategy depended on the nature of the antioxidant activity being assessed. Thus, for the inhibition of free radical formation and organic peroxide scavenging activities, blood serum possessed significantly less activity than plasma (Figure 2A,C; *p* < 0.05), while for hydrogen peroxide scavenging activity, the opposite was the case (Figure 2B; *p* < 0.05). However, no significant differences were observed between serum and plasma when assessing free radical scavenging activity.

### 3.3. Seminal Plasma in Different Species

**Figure 3:**
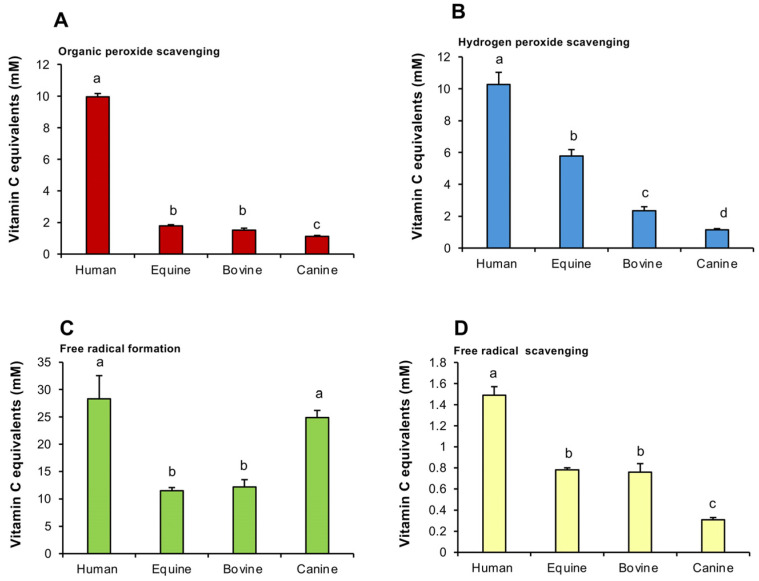
Antioxidant activity in the seminal plasma in different species. (**A**) Organic peroxide scavenging activity. (**B**) Hydrogen peroxide scavenging activity. (**C**) Inhibition of ABTS^•+^ radical formation. (**D**) ABTS^•+^ radical scavenging activity. All results are expressed as vitamin C equivalents. All columns not connected by the same letter are significantly different. Data presented as means ± S.E.M.; *n* = 9.

Following on from these experiments, a comparative analysis was undertaken profiling the antioxidant activity of SP from different species (human, equine, bovine and canine SP). The results of this analysis indicated that the ability of human plasma to scavenge organic peroxides was significantly greater than that of any of the other species, while equine and bovine SPs possessed significantly (*p* < 0.001) more activity than their canine counterpart (Figure 3A). Similarly, in terms of hydrogen peroxide scavenging activity, human SP possessed significantly (*p* < 0.001) greater activity than any of the other species examined, while equine SP was significantly greater than bovine, and both equine and bovine SPs were more active than the canine material (*p* < 0.001) (Figure 3B).

The ability of human SP to suppress free radical formation was also significantly greater than either bovine or equine material (Figure 3C; *p* < 0.001). However, with this assay, canine SP was just as active in the suppression of free radical formation as human SP and significantly greater than that observed with bovine or equine SP (Figure 3C; *p* < 0.001). Finally, the ability of human SP to scavenge free radicals was also higher (*p* < 0.001) than all of the other species examined, while both equine and bovine SP possessed more such activity than the canine equivalent (Figure 3D; *p* < 0.001). In general, the hierarchy of antioxidant protection observed in this comparative study was human > equine > bovine > canine, although the latter was surprisingly active in the suppression of free radical formation.

### 3.4. Impact of Oxidative Stress on Sperm Motility in Different Species

A possible explanation for the relatively high level of antioxidant protection afforded by human SP, particularly with respect to organic peroxide scavenging (Figure 2A), would be that human spermatozoa are particularly vulnerable to oxidative stress created by lipid peroxides. In order to test this hypothesis, spermatozoa from three different species were exposed to cumene hydroperoxide and the impact on sperm motility assessed using a CASA system.

**Figure 4:**
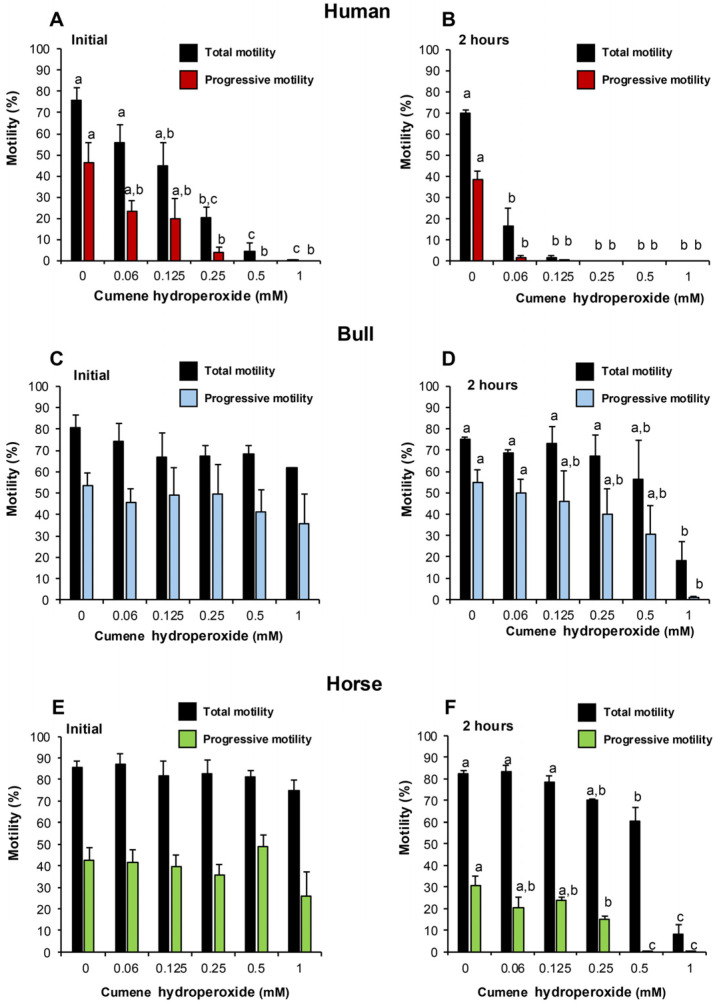
Dose-dependent impact of cumene hydroperoxide on sperm motility in different species. Both total motility and progressive motility were assessed using a CASA system and two time points were examined: 15–30 min and 2 h. (**A**) Human spermatozoa after 15–30 min exposure to cumene hydroperoxide. (**B**) Human spermatozoa after 2 h exposure to cumene hydroperoxide. (**C**) Bovine spermatozoa after 15–30 min exposure to cumene hydroperoxide. (**D**) Bovine spermatozoa after 2 h exposure to cumene hydroperoxide. (**E**) Equine spermatozoa after 15–30 min exposure to cumene hydroperoxide. (**F**) Equine spermatozoa after 2 h exposure to cumene hydroperoxide. All columns not connected by the same letter are significantly different. Data presented as means ± S.E.M.; *n* = 3.

As hypothesised, human spermatozoa were found to be very vulnerable to oxidative attack. Thus, an analysis of sperm movement within 15–30 min of adding peroxide, revealed that both total and progressive motility were significantly (*p* < 0.001) reduced relative to control levels at doses of cumene hydroperoxide above 0.25 mM (Figure 4A). In contrast, no significant impact on bovine or equine spermatozoa was recorded under identical conditions (Figure 4C,E). When the exposure time to cumene hydroperoxide was extended to 2 h, the total and progressive motility of human spermatozoa was significantly suppressed at doses above 0.06 mM (*p* < 0.001), while at doses greater than 0.25 mM, these cells were completely motionless (Figure 4B). In the case of bovine spermatozoa, however, neither the total nor the progressive motility of these cells was impacted by a 2 h exposure to cumene hydroperoxide except at the highest dose examined of 1 mM (*p* < 0.05; Figure 4D). Similarly, a 2 h exposure to cumene hydroperoxide did not impact the total motility of equine spermatozoa until doses of 0.5 mM (*p* < 0.01) and 1 mM (*p* < 0.001) were reached (Figure 4F), while progressive motility was not compromised until the cumene hydroperoxide dose exceeded 0.25 mM (*p* < 0.05; Figure 4F). Taken together, these data indicate a hierarchy of vulnerability such that human > equine > bovine, in keeping with the above analyses of SP antioxidant activity.

### 3.5. Impact of Seminal Plasma on Peroxide-Mediated Toxicity

**Figure 5:**
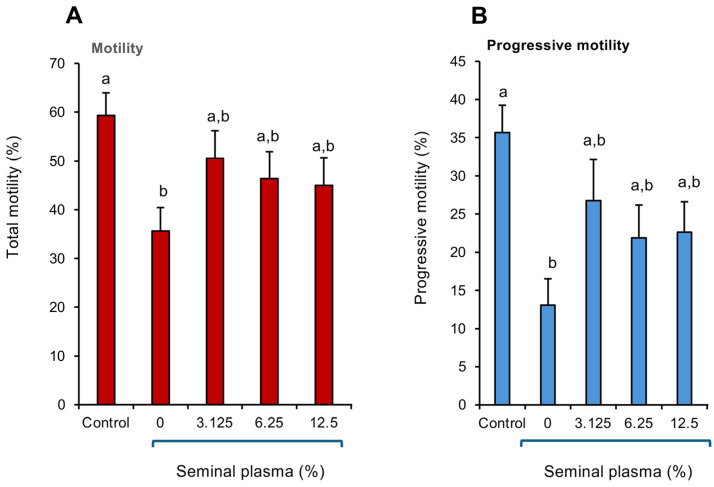
Analysis of the impact of human seminal plasma on the cytotoxic influenceof cumene hydroperoxide on human spermatozoa. A fixed concentration of purified human spermatozoa (10 × 10^6^/mL) was exposed to 0.25 mM cumene hydroperoxide alone or in the presence of human seminal plasma (3.125–12.5%) and the impact on sperm motility assessed using CASA. (**A**) Total motility. (**B**) Progressive motility. A 15–30 min exposure to cumene hydroperoxide significantly suppressed both motility (*p* < 0.05) and progressive motility (*p* < 0.01); however, this peroxide-mediated impact was negated by the presence of seminal plasma. All columns not connected by the same letter are significantly different. Data presented as means ± S.E.M.; *n* = 7.

In order to confirm the powerful antioxidant protection offered by human seminal plasma, a study was performed in which the spermatozoa were exposed to a fixed dose of cumene hydroperoxide (0.25 mM) in the presence or absence of human seminal plasma. Their functionality was then assessed with a focus on CASA measurements of total and progressive motility. This analysis revealed that exposure to 0.25 mM cumene peroxide precipitated the anticipated decline in both total (Figure 5A; *p* < 0.05) and progressive (Figure 5B; *p* < 0.01) motility within a 15–30 min time span (the time needed to conduct all of the CASA analyses). However, in the presence of as little as 3.125% human seminal plasma, this toxic oxidative impact was negated and neither the percentages of motile nor progressively motile cells were significantly different from the untreated control sample, as long as seminal plasma was present (Figure 5A,B).

## 4. Discussion

The data secured in this study demonstrate that the RoXsta^TM^ system is capable of rapidly and efficiently profiling the antioxidant activity in a wide range of different biological materials, including blood serum and plasma, follicular fluid, saliva, urine and semen. Additional studies have confirmed that this technology is capable of generating meaningful antioxidant data on other materials including fruit juices (Figure S1, Supplementary Materials) and even commercial cosmetic formulations (Figure S2, Supplementary Materials).

Of course, many assays for measuring antioxidant activity already exist, including those focused on free radical scavenging activity using either ABTS or 2,2-diphenyl-1-picrylhydrazyl (DPPH) as redox sensors, as well as analyses of ferric reducing antioxidant power (FRAP), cupric reducing antioxidant capacity (CUPRAC) and oxygen radical absorption capacity (ORAC), each assay having its own strengths and weaknesses [20]. Compared with all of these laboratory assays, the RoXsta^TM^ system possesses at least two distinct advantages: (i) it can measure several different types of antioxidant activity including free radical scavenging, hydrogen peroxide scavenging and organic peroxide scavenging activities, as well as the suppression of free radical formation [17] and (ii) the use of an electrochemical cell enables the free radical elements of this assay to be conducted within a matter of minutes (~5 min) with a minimum of laboratory equipment. Ultimately, the speed and efficiency of this system should permit its development as a point-of-care assay that will enable rapid assessments of antioxidant activity to be conducted in the immediate vicinity of the patient. In the following discussion, we consider (i) how the antioxidant profiles generated with this assay system vary according to the nature of the fluid under investigation, irrespective of the species of origin and (ii) how, in contrast, the antioxidant activity of seminal plasma shows dramatic interspecies variation depending on the relative vulnerability of the spermatozoa to oxidative attack.

### 4.1. Antioxidant Profiling of Different Biological Fluids

A clear limitation of this comparative study is that we were restricted to the biological fluids that were available to us, including equine blood plasma and serum, human saliva and semen and bovine follicular fluid and urine. Despite the use of different source species, the results are generally representative of the fluid in question. Thus, our free radical scavenging assay is broadly similar to the TAC assay (Cayman Chemical Co., Ann Arbor, MI, USA) originally developed by Rice-Evans and colleagues, except that the ABTS^•+^ radical is generated electrochemically rather than using the conventional potassium persulphate or metmyoglobin/H_2_O_2_ ABTS oxidation strategy [21]. Despite these methodological differences, our assessment of the antioxidant potential of human seminal plasma (Figure 2D) or intact semen (Figure 2D) using the free radical scavenging assay (~1.4 mM vitamin C equivalents) agrees extremely well with the value of 1.4 mM Trolox equivalents generated by Mahfouz et al. [22] using the conventional TAC assay. Similarly, our estimation of salivary antioxidant levels using the free radical scavenging assay (~0.22 mM vitamin C equivalents) approximates to published values for human saliva acquired using the traditional ABTS assay (0.34 ± 0.037 mM Trolox equivalents) [23] and agrees well with similar estimates made in pigs (~0.3–0.4 mM Trolox equivalents) [24]. In terms of blood, our estimates of the free radical scavenging activity of equine plasma and serum (~0.41 and 0.39 mM vitamin C equivalents, respectively) agrees with previous assessments of equine blood plasma (~0.37–0.40 mM Trolox equivalents) using the ABTS-based TAC assay [25] and reflects similar determinations in dog (0.312 ± 0.015 mM Trolox equivalents) [26], cattle (~0.12 mM Trolox equivalents) [27] and humans (0.29 mM Trolox equivalents) [28]. Similarly, the intense antioxidant activity measured in bovine urine with the free radical scavenging assay (~4 mM Vitamin C equivalents) reflects the high values recorded in human (3.13 ± 0.11 mM Trolox equivalents) with ABTS free radical scavenging assays [29] and comparative studies conducted using alternative methods to measure total antioxidant activity in human body fluids [30]. Our finding that follicular fluid possesses a relatively low free radical scavenging capacity (generally < 1.5 mM) compared with semen and urine also resonates with previous analyses on water buffalo [31], humans [32] and cattle [33].

The high antioxidant activity expressed by urine in the electrochemical determinations of free radical formation and scavenging may reflect the mediation of hydroxyl radicals in these assays, in keeping with the known capacity of uric acid to scavenge this radical species [34,35]. In contrast, urine was relatively less active in the hydrogen peroxide scavenging assay, possibly because uric acid reacts poorly with this oxidant [36]. Blood, plasma and serum were characterized by broadly similar levels of antioxidant activity, although the inhibition of free radical formation assay revealed significantly higher levels of activity in plasma than serum (Figure 2C). Evidently, the factors responsible for suppressing formation of the ABTS^•+^ radical in an electrochemical cell can be compromised by the clotting procedures used to generate serum.

### 4.2. Comparative Analysis of Antioxidant Profiles in Seminal Plasma of Different Species

Notwithstanding the significant antioxidant activity present in blood and urine, the most powerful antioxidant activity was consistently observed in human semen. In order to determine whether this was a unique feature of the human ejaculate or a general characteristic of SP, we examined semen samples from four different sources (human, bovine, equine and canine) and revealed hitherto unknown, and unsuspected, interspecies differences in the level and type of antioxidant protection afforded by this fluid. In general, human seminal plasma was shown to possess the greatest antioxidant potential, particularly in terms of its organic peroxide and hydrogen peroxide scavenging activities. This observation accords with existing data indicating that human spermatozoa are particularly susceptible to the toxic impact of lipid peroxides and that this impact can be reversed by the addition of SP [37]. These data also accord with a large number of studies indicating that the antioxidant capacity of human seminal plasma is significantly lower in cases of male infertility and is inversely correlated with the accumulation of lipid peroxidation markers such as malondialdehyde [38,39]. Analyses of the TAC values associated with human semen have also shown positive correlations with multiple parameters of semen quality, including sperm motility, concentration, morphology and DNA integrity [40,41,42], while being negatively associated with measurements of lipid peroxidation [38]. Furthermore, the losses of sperm function associated with clinical conditions such as varicocele [43], environmental exposures to air pollutants and glyphosate [44], as well as the adverse impact of lifestyle factors, including cigarette smoking, diabetes and obesity [45,46], are all associated with decreases in the total antioxidant protection offered by SP.

Most of these studies have been conducted using the ABTS-based TAC assay and therefore reflect the ability of SP to scavenge the ABTS^•+^ radical. However, others have also measured hydrogen peroxide scavenging activity in assays exploiting the HRP-mediated oxidation of luminol [47]. Recently, electron spin resonance spectroscopy was used to monitor the radical scavenging activity of seminal plasma from multiple species (stallion, bull, man and lion) [48]. This analysis also revealed that the free radical scavenging capacity of human SP was more than ten times greater than the other species examined, in accord with the data presented in the present analysis [48]. Furthermore, it indicated the presence of significant free radical scavenging activity in bovine SP, in keeping with the present study and the results obtained by others, who detected antioxidant activity in bovine ejaculates that exhibited the anticipated negative correlation with lipid peroxidation status [49]. However, the study by Jakop et al. [48] failed to detect significant antioxidant activity in equine SP, in contrast to our data and the results of others who have found significant free radical scavenging activity in equine SP that had a significant impact on sperm function and DNA integrity [50]. For its part, canine SP has also been shown to possess a capacity for ABTS^•+^ radical scavenging by our group and others [51]. However, this is the first study to reveal the relative paucity of this activity in canine semen compared with other species in terms of its organic peroxide, hydrogen peroxide and free radical scavenging activity. It is also the first to reveal that canine SP possesses a particularly high capacity to suppress free radical formation (Figure 3C).

The relatively high level of antioxidant protection afforded by human SP emphasises the importance that evolution has attached to protecting human spermatozoa from oxidative stress. In light of these findings, the RoXsta^TM^ system may be of value in rapidly diagnosing antioxidant deficiencies within the patient population and identifying those individuals for whom antioxidant therapy represents a rational therapeutic option. [16]. Interestingly, the egg is not offered the same high level of antioxidant protection given the relatively modest levels of antioxidant activity detected in follicular fluid, which was not fundamentally higher than blood plasma, with the exception of the inhibition of free radical formation, where it was lower (Figure 2C). Although relatively low, the levels of free radical scavenging activity measured in follicular fluid correspond with those observed in clinical studies (~0.3–0.7 mM), which have, in turn, been correlated with the incidence of pregnancy in assisted conception cycles [52].

The need for greater extracellular antioxidant protection for spermatozoa compared with oocytes is entirely explicable on the basis of cell architecture. Spermatozoa possess an extremely limited cytoplasmic space in terms of absolute volume and restricted location within the sperm midpiece, as a result of which there is little space in which to house the antioxidant factors that protect most cells from oxidative stress. Consequently, these cells are uniquely dependent on the availability of antioxidants in the extracellular space during their passage from the germinal epithelium of the testes to the ampulla of the fallopian tubes, where fertilization occurs. It is for this reason that SP, as well as the spaces inhabited by spermatozoa within the testes, epididymis, and female tract, are so well endowed with extracellular antioxidants [53,54,55]. By contrast, the oocyte possesses significant cytoplasmic volume in which to sequester cellular antioxidants and, in any case, is metabolically dormant for much of its life, surrounded by a protective cocoon of pre-granulosa cells within the primordial follicle [56]. However, as soon as the oocyte starts to grow and mature within the Graafian follicle, it becomes vulnerable to oxidative stress and dependent on the antioxidant protection afforded by follicular fluid [57,58], even if such protection does not approach the level of antioxidant protection offered to spermatozoa by SP.

The particularly high levels of antioxidant protection that characterize human SP in comparison to other species reflects the particular vulnerability of human spermatozoa to peroxide attack as revealed in Figure 4A–F and Figure 5A,B. This vulnerability may stem from differences in the energy metabolism exhibited by the spermatozoa of different species. Thus canine, equine and bovine spermatozoa are heavy users of oxidative phosphorylation [59,60]. As a consequence, ROS generation is a normal feature of sperm metabolism in these species to the extent that positive correlations have been observed between sperm motility and intracellular ROS generation [60,61]. Given this inherently high level of exposure to ROS, mechanisms have evolved to provide some level of resistance against oxidative stress in those species that exploit the high levels of ATP generation associated with intense mitochondrial respiration [62]. By contrast, human spermatozoa are built more for distance than for speed. This strategy may have evolved because our species is unusual in that insemination is not synchronized with ovulation through the mediation of behavioural oestrus. As a result, human spermatozoa may have to survive in the female tract for several days waiting for an egg to be released from the ovary. To achieve this longevity, human spermatozoa have developed a dependency on glycolysis, sacrificing the ATP-generating capacity of oxidative phosphorylation for a low level of exposure to mitochondrial ROS [63,64]. This lack of exposure to intracellular ROS has created a dependency on antioxidant protection in the extracellular space, to defend these cells against oxidative stress; hence the unusually high levels of antioxidant protection offered by human SP, and its diagnostic significance.

## 5. Conclusions

In conclusion, the RoXsta^TM^ system appears to be a convenient, rapid method of measuring and characterising antioxidant activity in a wide variety of clinical and commercial contexts. The simplicity and rapidity of the RoXsta^TM^ system should be of value to clinicians since it provides an opportunity for real-time, point-of-care assessments to diagnose those patients suffering from oxidative stress and to monitor the effectiveness of antioxidant supplementation in restoring an appropriate redox balance. Such assessments may be particularly important in the field of male infertility where deficiencies in the levels of antioxidant afforded to the spermatozoa have been clearly, and causally, linked to the impairment of human sperm function.

## 6. Patents

The methodology described in the article is subject to a patent application (PCT/AU2024/050943).

## Data Availability

All data are contained within the article.

## Supplementary Materials

The following supporting information can be downloaded at: https://www.mdpi.com/article/10.3390/antiox14010090/s1, Table S1: Optimised sample dilutions for antioxidant assays; Figure S1: Ability of the RoXsta^TM^ system to profile the antioxidant activity of fruit juices; Figure S2: Ability of the RoXsta^TM^ system to profile the antioxidant activity of cosmetic products [65,66].
