# Supplementary material for: A Comparative Analysis of the Antioxidant Profiles Generated by the RoXstaTM System for Diverse Biological Fluids Highlights the Powerful Protective Role of Human Seminal Plasma
Source: Antioxidants (Basel). 2025 Jan 14;14(1):90. doi: 10.3390/antiox14010090 (PMC11762656; doi:10.3390/antiox14010090)
Supplement: Supplementary file 1 [file antioxidants-14-00090-s001.zip › Supplementary Figure S1 Ability of the RoXsta system to profile the antioxidant activity of fruit juices.pdf]

Supplementary Figure S1

Ability of the RoXsta™ system to profile the antioxidant activity of fruit juices

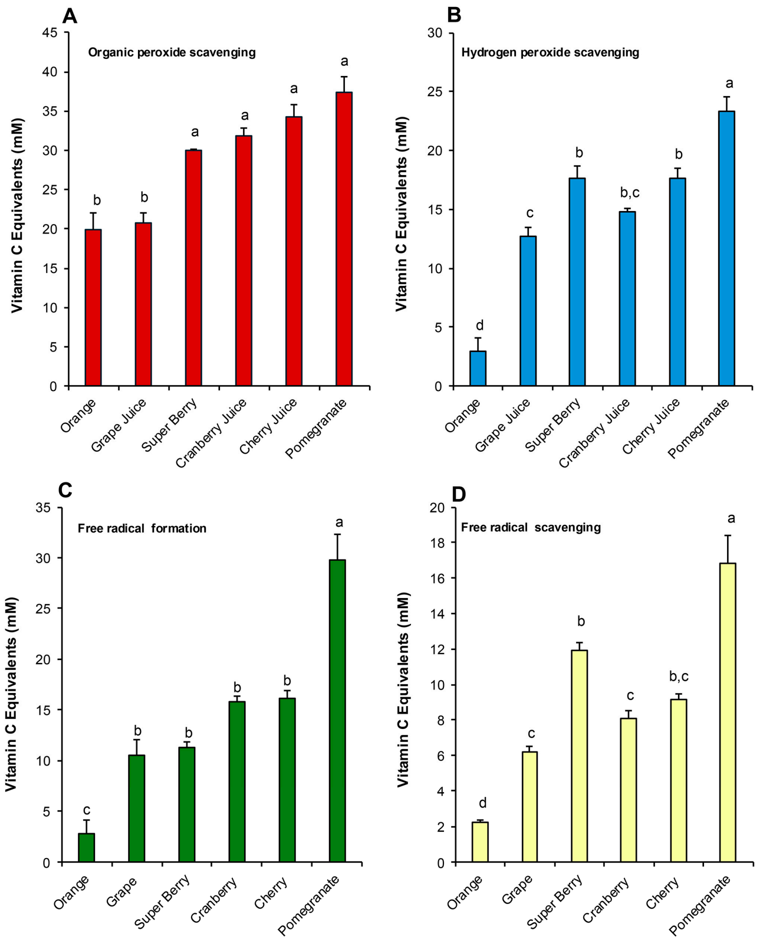

Supplementary Figure S1. Analysis of antioxidant activity in a range of fruit juices using the RoXsta™ system. (**A**) Organic peroxide scavenging activity. (**B**) Hydrogen peroxide scavenging activity. (**C**) Inhibition of ABTS$^{\bullet+}$ radical formation. (**D**) ABTS$^{\bullet+}$ radical scavenging activity. All results are expressed as vitamin C equivalents. Statistical analysis by ANOVA following Box-Cox transformation; data presented as means ± SEM (*n* = 3). All columns not connected by the same letter are significantly different.

For these analyses various fruit juices were purchased from a local supermarket to test for antioxidant activity: cranberry (OceanSpray, Lakeville-Middleboro, MA, USA), cherry (Bickfords, Salisbury South, SA, Australia), grape (Bickfords), pomegranate (Bickfords), superberry (an antioxidant blend of red grapes, pomegranates, cranberries, strawberries and raspberries; Bickfords) and orange (Nudie – nothing but orange juice with pulp, Eastgardens, NSW, Australia).

With all four variants of the assay system, pomegranate juice was shown to exhibit the highest activity, while grape and orange juice exhibited the lowest. Super-berry juice was evidently a very good scavenger of free radicals and hydrogen peroxide, while cherry juice was an excellent scavenger of organic peroxides. These results are in keeping with previous comparative studies that have also demonstrated the relative superiority of pomegranate juice as an antioxidant using a variety of assays that largely reflect its high phenolic content [1,2].

References

1. Seeram, N.P.; Aviram, M.; Zhang, Y.; Henning, S.M.; Feng, L.; Dreher, M.; Heber, D. Comparison of antioxidant potency of commonly consumed polyphenol-rich beverages in the United States. *J. Agric. Food Chem.* **2008**, 56, 1415-1422.

2. Granato, D.; Karnopp, A.R.; van Ruth, SM. Characterization and comparison of phenolic composition; antioxidant capacity and instrumental taste profile of juices from different botanical origins. *J. Sci. Food Agric*. **2015**, 95, 1997-2006.
