# Supplementary material for: A Comparative Analysis of the Antioxidant Profiles Generated by the RoXstaTM System for Diverse Biological Fluids Highlights the Powerful Protective Role of Human Seminal Plasma
Source: Antioxidants (Basel). 2025 Jan 14;14(1):90. doi: 10.3390/antiox14010090 (PMC11762656; doi:10.3390/antiox14010090)
Supplement: Supplementary file 1 [file antioxidants-14-00090-s001.zip › Supplementary Figure S2 Ability of the RoXsta system to profile the antioxidant activity of cosmetic products .pdf]

Supplementary Figure S2

Ability of the RoXsta™ system to profile the antioxidant activity of cosmetic products

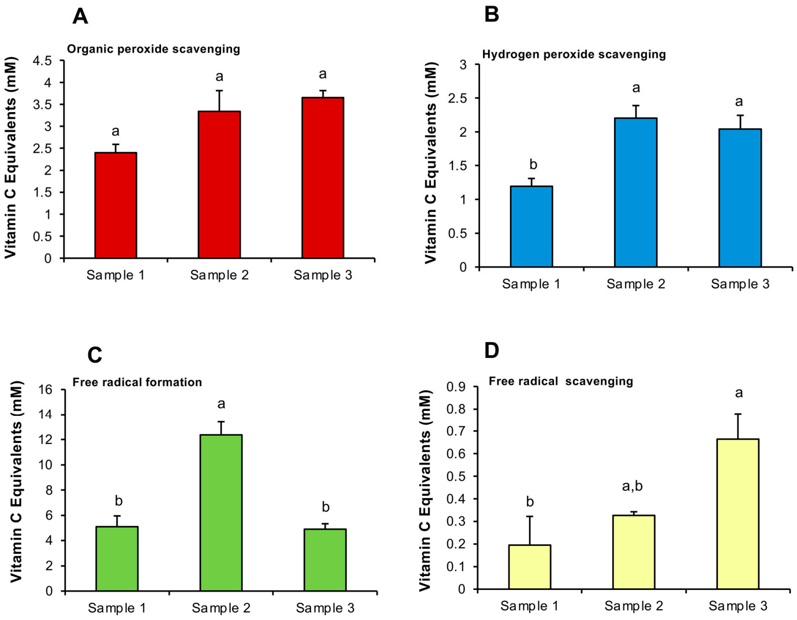

Supplementary Figure S2. Analysis of antioxidant activity in a range of cosmetic skin sera using the RoXsta™ system. (**A**) Organic peroxide scavenging activity. (**B**) Hydrogen peroxide scavenging activity. (**C**) Inhibition of $ABTS^{•+}$ radical formation. (**D**) $ABTS^{•+}$ radical scavenging activity. All results are expressed as vitamin C equivalents. Statistical analysis by ANOVA following Box-Cox transformation; data presented as means ± SEM (n = 3). All columns not connected by the same letter are significantly different.

For these analyses, Water-based cosmetic serums were purchased from a retailer for the assessment of antioxidant activity in skincare products and included: (1) Mecca Cosmetica Replenishing Niacinamide Serum (Mecca, Melbourne, VIC, Australia), (2) Estée Lauder Advanced Night Repair, (Estée Lauder Inc, NY, USA) and (3) Dermalogica Dynamic Retinol Serum (Dermatological Carson, CA, USA).

The results of this analysis showed that the RoXsta™ system was able to generate antioxidant profiles that effectively differentiated different commercial products. Thus, while all three sera were equally active as scavengers of organic peroxides (Supplementary Figure S2A), Samples 2 and 3 were significantly ($p < 0.01$) more active in scavenging hydrogen peroxide than Sample 1 (Supplementary Figure S2B). However, in terms of the suppression of free radical formation, Sample 2 was the most active ($p < 0.01$) (Supplementary Figure S2C), whereas Sample 3 was the most effective free radical scavenger ($p < 0.05$) (Supplementary Figure S2D).

Further studies will be needed to determine whether one form of antioxidant activity (free radical scavenging, organic peroxide scavenging etc) is preferred over another in terms of delivering effective skin care.
