# Supplementary material for: A Comparative Analysis of the Antioxidant Profiles Generated by the RoXstaTM System for Diverse Biological Fluids Highlights the Powerful Protective Role of Human Seminal Plasma
Source: Antioxidants (Basel). 2025 Jan 14;14(1):90. doi: 10.3390/antiox14010090 (PMC11762656; doi:10.3390/antiox14010090)
Supplement: Supplementary file 1 [file antioxidants-14-00090-s001.zip › Supplementary Table S1 Optimised sample dilutions for antioxidant assays .pdf]

Supplementary Table S1. Optimised sample dilutions for antioxidant assays

	Organic Peroxide Scavenging (in 100µL)	Hydrogen Peroxide Scavenging (in 100µL)	Free radical scavenging (to 1 ml ABTS$^{\bullet+}$)	Inhibition of ABTS$^{\bullet+}$ formation (in 100µL)
**Semen (Human)**	1.5%	1.5%	15 µl at 100%	8.25%
**Saliva (Human)**	10%	30%	80 µl at 100%	50%
**Blood Plasma (Equine)**	20%	10%	30 µl at 100%	8.25%
**Blood Serum (Equine)**	20%	10%	30 µl at 100%	8.25%
**Urine (Bovine)**	2.5%	2.5%	15 µl at 25%	3%
**Follicular Fluid (Bovine)**	20%	15%	30 µl at 100%	20%
**Fruit Juice**	0.5%	1%	15 µl at 10%	15%
**Cosmetics (Serum)**	10%	10%	50 µl at 50%	30%
**Seminal Plasma (Human)**	1.5%	1.5%	15 µl at 100%	8.25%
**Seminal Plasma (Equine)**	10%	5%	30 µl at 100%	20%
**Seminal Plasma (Bovine)**	15%	10%	15 µl at 100%	15%
**Seminal Plasma (Canine)**	30%	15%	30 µl at 100%	15%
